# Type 2 Diabetes Mellitus Related Distress in Thailand

**DOI:** 10.3390/ijerph17072329

**Published:** 2020-03-30

**Authors:** Kongprai Tunsuchart, Peerasak Lerttrakarnnon, Kriengkrai Srithanaviboonchai, Surinporn Likhitsathian, Sombat Skulphan

**Affiliations:** 1 Department of Community Medicine, Faculty of Medicine, Chiang Mai University, Chiang Mai 50200, Thailand or ksrithanaviboonchai@gmail.com (K.S.); 2 Department of Family Medicine, Faculty of Medicine, Chiang Mai University, Chiang Mai 50200, Thailand; 3 Research Institute for Health Sciences, Chiang Mai University, Chiang Mai 50200, Thailand; 4 Department of Psychiatry, Faculty of Medicine, Chiang Mai University, Chiang Mai 50200, Thailand or; 5 Department of Psychiatric Nursing, Faculty of Nursing, Chiang Mai University, Chiang Mai 50200, Thailand; sombat.sk@cmu.ac.th

**Keywords:** diabetes-related distress (DRD), type 2 diabetes mellitus, primary care

## Abstract

This study aimed to investigate prevalence and factors potentially associated with diabetes-related distress (DRD) among type 2 diabetes mellitus (T2DM) patients in a primary health care center in Thailand. This cross-sectional study was conducted with a total of 370 patients with T2DM. Data were collected at primary health care centers in Hang Dong District, Chiang Mai Province, Thailand. DRD was assessed using the Diabetes Distress Scale (DDS-17). The association between sociodemographic characteristics and other factors with DRD was analyzed using the Fisher t-test, Chi-square test, and Pearson’s correlation coefficient test. The association between Hemoglobin A1c (HbA1c) and DRD was analyzed using multiple linear regression analysis. The participants had a mean age of 60.95 ± 7.96, and most were female (68.1%). Of the participants with DRD, 8.9% had moderate to high levels of distress. Education level and family support were significantly associated with the overall level of DRD. Additionally, HbA1c and co-morbidity were also significantly associated with DRD, as were emotional burden and regimen distress. Multiple linear regression analysis found that increased HbA1c was positively associated with increased DRD after adjusting for age, sex, education, duration of T2DM, co-morbidity, diabetic complications, and family support. Screening with DRD may be beneficial in T2DM patients.

## 1. Introduction

Diabetes mellitus (DM) is a chronic disease, and one of the most common metabolic disorders globally. The International Diabetes Federation (IDF) reported in 2019 that 436 million adults were living with diabetes around the world, and that this number was projected to increase to 700 million by 2045 [1]. Approximately 90% of the total were type 2 DM (T2DM) [1]. Thailand has the seventh largest diabetic patient population in the Western Pacific [2], with a prevalence of 9.9% in the adult population [3]. Diabetes is a leading cause of death in Thailand. Of the more than 200,000 deaths annually due to chronic non-communicable diseases in Thailand, approximately 30,000 (15%) are due to diabetes [4].

Diabetes mellitus is a condition in which the body has high blood sugar levels (hyperglycemia) due to a lack of insulin hormones, impaired secretion of insulin, or both [5]. Prolonged high blood sugar levels can result in the destruction, deterioration, and failure of vital organs [6]. Inability to control blood sugar levels can result from both non-modifiable factors and from modifiable factors. Non-modifiable factors include gender, age, duration of diabetes, microvascular and macrovascular complications, and comorbidity [7,8]. Modifiable factors include behavioral and/or psychosocial issues, e.g., incorrect eating habits, not exercising, obesity or high body mass index, not consulting with a doctor, and not taking medication as prescribed by a doctor [9,10,11,12,13], as well as stress, depression and anxiety [14,15], especially diabetes-related distress [16,17,18].

Diabetes-related distress (DRD) is a syndrome comprised of multidimensional components including worry, conflict, frustration, and discouragement that can accompany living with diabetes. Negative physical and psychological effects can be directly attributable to long-term suffering from diabetes-related emotional distress [19,20]. Sixty percent of patients with diabetes who have high levels of negative mood and/or psychological distress display a high level of distress [21]. Previous studies have reported a high prevalence of DRD in individuals with T2DM, e.g., the prevalence was 64% in China [22], 63.7% in Iran [23], 49.2% in Malaysia [24], and 48.5% in Bangladesh [25]. Moreover, previous studies have reported that long-term DRD in diabetic patients is associated with anxiety [26], depression [22,27,28,29], low levels of physical activity [18], obesity [28], low medication compliance [30,31], poor diet control, low self-efficacy [32], poor self-care behavior [21], and poor quality of life [33]. A high level of DRD has also been shown to be associated with high HbA1c levels [17,18,30]. Furthermore, the bidirectional association between chronic physical diseases (CPD) and psychopathological factors might lead to an exacerbation of both conditions [34].

Most clinicians are aware that emotional distress is common among their patients with diabetes and that this distress has a deleterious effect on diabetes outcomes; however, many clinicians feel unable to treat this distress [35]. Nevertheless, the health consequences of emotional problems are associated with poor self-care behavior, poor metabolic outcomes, morbidity, mortality, functional limitations, and reduced quality of life [35]. These negative effects are not limited to diagnosable psychiatric disorders [21]. Thus, addressing these emotional problems is key to successful health care intervention, even if diabetes self-care is adequate [35]. For that reason, DRD screening is an important first step in the treatment of mental health problems of patients with T2DM. In Thailand, there have been few studies of the prevalence of DRD and factors associated with DRD in T2DM. This study aimed to investigate prevalence and factors associated with DRD among T2DM.

## 2. Materials and Methods

### 2.1. Study Design, Population

This cross-sectional study of 370 respondents from a total DM population of 3894 in Hang Dong District, Chiang Mai Province, Thailand, was conducted between January and March 2017 at thirteen primary health care centers in Hang Dong District. Inclusion criteria were being a patient with type 2 DM, aged 18 years or above. Exclusion criteria were inability to communicate in the Thai language, communication disorder, a history of psychiatric disorders, Alzheimer’s disease, and dementia. 

Respondents were chosen by simple random sampling. Sample size was calculated using the n4Studies application for estimating the proportion of a finite population [25] and was based on the 48.5% of DRD; delta = 0.05, alpha = 0.05, Z (0.975) = 1.960 patients in the study area eligible for inclusion in the analysis [36]. The formula is defined follow:

(1) $$n=\frac{Np\left(1-p\right)z_{1-\frac{\alpha }{2}}^{2}}{d^{2}\left(N-1\right)+p\left(1-p\right)z_{1-\frac{\alpha }{2}}^{2}}$$

### 2.2. Data Collection and Diabetes-Related Distress Level Measurement

Respondents were recruited into the study after providing written informed consent. All subjects were interviewed to obtain demographic information (age, sex, marital status, education, occupation, family support, and medical history (diabetic complications, co-morbidity, duration of DM, type of diabetic management, HbA1c level).

The Diabetes Distress Scale (DDS-17) questionnaire for assessing DRD developed by Polonsky et al. [37] was translated into the Thai language by Kattika [20]. The alpha coefficient of the questionnaire was 0.95. The questionnaire is composed of 17 items grouped in four sub-components: five items about emotional burden (EB), three items about regimen-related distress (RD), three items about interpersonal-related distress (ID), and four items about physician-related distress (PD). Each item is rated on a 6-point scale from 1 (no problem) to 6 (serious problems). Sub-components were interpreted as the mean score of the sub-component: <2 = little or no distress, 2–2.9 = moderate distress, and ≥3 = a high level of distress.

### 2.3. Statistical Analysis

Descriptive statistics are reported as frequencies, means, and standard deviations. The associations between sociodemographic characteristics, potentially related factors, and DRD were analyzed using the Fisher t-test, Chi-square test, and Pearson’s correlation coefficient test. The association between HbA1c level and DRD was analyzed using multiple linear regression analysis.

### 2.4. Ethical Considerations

The study was approved by the Research Ethics Committee of the Faculty of Medicine, Chiang Mai University, Thailand (REF: COM-2016-05965).

## 3. Results

The average age of respondents was 60.95 years (±7.96); most were female (68.1%), married (69.8%), had at least some formal education (90.8%), were currently employed (69.7%). A family member was the primary caretaker in most cases (76.8%). Most of the patients had complications (84.9%) and comorbidity (81.9%). An average duration of T2DM was 9.17 ± 6.46 years. The most frequent treatment method was medication (95.1%). The mean HbA1c level was 8.02 ± 1.55% (Table 1).

Of the DRD cases identified by DDS-17, 1.1% were found to be at a high level, 7.8% at a moderate level, and 91.1% at a mild level. The EB sub-component had the greatest moderate or high level of DRD (27.1%). Physician-related distress had the least moderate plus high level of DRD (4.3%) (Table 2).

This study found that education (*p* < 0.05) and family support (*p* < 0.05) were significantly associated with the level of total diabetes-related distress. However, age, sex, marital status, occupation, diabetic complications, co-morbidity, duration of DM, type of diabetic management, and HbA1c were not significantly associated with the level of total DRD (Table 3).

The study found that HbA1c and co-morbidity were significantly associated with total DRD, EB, and RD. However, HbA1c and co-morbidity were not significantly associated with PD or ID. In addition, age and duration of DM were not significantly associated with total DRD, EB, PD, RD, or ID. (Table 4)

Multiple linear regression analysis found that higher HbA1c level was positively associated with increasing greater diabetes-related distress after adjusting for age, sex, education, duration of DM, co-morbidity, diabetic complications, and family support (Table 5).

## 4. Discussion

This study aimed to assess prevalence of DRD, and factors potentially associated with T2DM at the primary care level. Prevalence of DRD from DDS-17 was 8.9%, of which 1.1% was at the high level and 7.8% at the moderate level. That is lower than other studies, e.g., the prevalence of DRD was 49.2% in Malaysia [24], 48.5% in Bangladesh [25], 42.15% in China [38], 51.3% in the US [16], and 63.7% in Iran [23]. Conversely, a study in Germany conducted at a tertiary care facility reported the prevalence of DRD was 8.9% using the Problem Area in Diabetes (PAID) questionnaire, the same level found in this study. However, another German study conducted at the primary care level reported only a 1.2% prevalence of DRD using the PAID scale [39,40]. The differences in DRD prevalence might result from differences in the assessment tools, i.e., the PAID and the DDS-17 questionnaires, which could potentially affect prevalence, e.g., sample size, access to health care, care setting [39], and demographic variables, e.g., education level and family support, as well as health status, e.g., HbA1c level, and co-morbidity [24,41,42]. The findings of these studies are consistent with our finding that DRD prevalence is higher in people with type 2 diabetes treated in a secondary and tertiary care setting than in those treated in a primary care setting [39,43]. 

Our study found that education level and family support were both significantly associated with the level of total diabetes-related distress (p = 0.007 and p = 0.037, respectively). A previous study reported that the major predictor for high diabetes distress scores among diabetic cases was low education level [44]. That association may be the result of low education leading to poor knowledge about the illness and its complications, which, in turn, increases the risk of poor dietary habits, poor medication compliance, and fewer health check-ups. Another study found lack of supportive family behavior was positively associated with emotional distress [45]. When family members behave negatively, e.g., by nagging or criticizing specific health-related behaviors, people with type 2 diabetes may respond by perceiving higher levels of diabetes-related distress. However, age, sex, marital status, occupation, diabetic complications, presence of co-morbidity, duration of DM, type of diabetic management, and HbA1c level (good, fair, poor) were not significantly associated with the level of total diabetes-related distress. The results of the present study are consistent with those of Zhou et al., which reported that the total DDS score was not significantly related to patients’ gender, age, diabetes duration, diabetes education, or complications [38]. On the other hand, other studies have reported that DRD is associated with age, duration of diabetes, occupation, marital status, glycemic status, treatment modalities, comorbidity, and diabetic complications [25,41,42]. These findings suggest that there may be other factors which affect DRD, e.g., type of diabetes, treatment regimen, and socioeconomic factors [21]. 

Total DRD, EB, and RD were found to be associated with both HbA1C and co-morbidity. EB includes a variety of negative emotions such as despair, conflict and fear-induced anger that result from thinking about the prospect of a lifetime of living with diabetes, and feeling overwhelmed by the many resulting demands [20]. Type D personality [46] and alexithymia [47] have been reported to adversely affect outcomes in patients with T2DM. However, our study did not attempt to identify those personality traits in the respondents. Further assessment of T2DM patients is needed to identify these personality traits in patients, and to measure the emotional burden they create. Appropriate emotional management of the EB dimension could assist in the control of blood sugar level [47]. RD encompasses concerns and discouragement that patients perceive and/or encounter while trying to self-manage their disease [20]. Integration of compliance, adherence, concordance, self-management, and empowerment could potentially improve medical approaches for RD patients [48]. In addition, age and duration of DM were not significantly associated with DRD, EB, PD, RD, or ID. Previous studies have reported that DRD is associated with HbA1c [42], that EB is associated with high HbA1c levels [49], and that RD is associated with high HbA1c values in hospital [50]. The present study found that both HbA1c and co-morbidity are associated with DRD. 

Multiple linear regression analysis found that higher HbA1c level is positively associated with increased diabetes-related distress after adjusting age, sex, education, duration of DM, co-morbidity, diabetic complications, and family support. These findings are consistent with a study by Tsujii et al. [17] that showed the association between glycemic control and diabetes distress among Japanese patients with type 2 diabetes. That study found diabetes distress was significantly associated with higher HbA1c levels. The relative risk for poor glycemic control (HbA1c ≥ 64 mmol⁄mol) was 8.0% when adjusted for age, sex, BMI, type of diabetes therapy, and duration of diabetes. A longitudinal study by Fisher et al. found the effect of diabetes distress in adults with type 2 diabetes that HbA1c was positively correlated with DRD [26]. Another study by Fisher et al., of the pattern of relationships between diabetic patients, used the DDS-17 to create a cut score for suffering among patients with type 2 diabetes. They found a consistent curvilinear relationship between the DDS and HbA1c [32]. A study by Tol et al., which assessed diabetes distress and related factors among type 2 diabetic patients to better tailor intervention planning in Isfahan, Iran, found diabetes-related distress in type 2 diabetic patients had a direct linear relation with HbAlc (r = 0.63, p < 0.001) [42]. Nanayakkara et al. explored the prevalence of, and factors associated with, diabetes distress in adults with type 2 diabetes in Australia. That study found diabetes distress was associated with higher HbA1c [50]. In addition, previous studies report psychological changes that occur with people with DM, which can have a significant impact on metabolic control. One of the major obstacles in this area is the need for a better understanding of psychological changes and their influence on changes in blood sugar levels [51].

Strengths of the present study include the use of a validated Thai language DDS-17 and an adequate sample size. A limitation of the study is that it was cross-sectional. Future studies should use a cohort methodology to confirm the causal relationship between HbA1c and DRD. Diseases were not classified in variables including complication and co-morbidity, which were confounding factors. Future studies should have the classification of co-morbidity and complication in order to control confounding factors which can affect DRD.

## 5. Conclusions

The prevalence of DRD is low at the primary care level. Education and family support are associated with DRD level. HbA1c level and co-morbidity are associated with the emotional burden and regimen-related distress sub-components of DRD. Screening with DRD may be beneficial in T2DM patients for reducing emotional problems, especially in patients with a low education level and patients with little or no family support. Further DRD studies should be conducted in secondary care and tertiary care settings.

## Figures and Tables

**Table 1:** Demographics characteristics of respondents (n = 370).

Characteristics (mean ± SD)	Clinical Sample n (%)
Age (years) (60.95 ± 7.96)	
<60	157 (42.4%)
≥60	213 (57.6%)
Sex	
Male	188 (31.9%)
Female	252 (68.1%)
Marital status	
Single	22 (5.9%)
Married	258 (69.8%)
Separated/Widowed	90 (24.3%)
Education	
None	34 (9.2%)
Some formal education	336 (90.8%)
Occupation	
Unemployed	112 (30.3%)
Employed	258 (69.7%)
Family support	
No	86 (23.2%)
Yes	284 (76.8%)
Diabetic complications	
Absent	56 (15.1%)
Present	314 (84.9%)
Co-morbidity	
Absent	67 (18.1%)
Present	303 (81.9%)
Duration of T2DM (years) (9.17 ± 6.46)	
0–10	256 (69.2%)
>10	114 (30.8%)
Type of diabetes management	
Lifestyle modification	7 (1.9%)
Oral hypoglycemic drugs	352 (95.1%)
Insulin use	7 (1.9%)
Insulin plus oral hypoglycemic drugs	4 (1.1%)
HbA1c (%) (8.02 ± 1.55)	
Good <7	90 (24.3%)
Fair 7–8	113 (30.5%)
Poor >8	167 (45.1%)

**Table 2:** Diabetes-related Distress of respondents (n = 370) by Diabetes Distress Scale (DDS)-17 level.

Domain of Distress	Level of Diabetes-Related Distress n (%)		
	Non or Mild	Moderate	High
Emotional burden (EB)	270 (73.0%)	82 (22.2%)	18 (4.9%)
Physician-related distress (PD)	354 (95.7%)	14 (3.8%)	2 (0.5%)
Regimen-related distress (RD)	313 (84.6%)	50 (13.5%)	7 (1.9%)
Diabetes-related interpersonal distress (ID)	324 (87.6%)	36 (9.7%)	10 (2.7%)
Total diabetes-related distress	337 (91.1%)	29 (7.8%)	4 (1.1%)

**Table 3:** Sociodemographic of respondents and level of diabetes distress (n = 370).

Characteristics	Level of Total Diabetes-Related Distress (%)			*p*-Value
	Non or Mild	Moderate	High	
Age, mean ± SD	60.9 ± 8.3	61.0 ± 5.7	65.0 ± 4.4	
<60	146 (43.3)	11 (37.9)	0 (0.0)	0.192
≥60	191 (56.7)	18 (62.1)	4 (100.0)	
Sex^b^				
Male	108 (32.0)	10 (34.5)	0(0.0)	0.374
Female	229 (68.0)	19 (65.5)	4 (100.0)	
Marital status				
Married	238 (70.6)	19 (65.5)	2 (50.0)	0.630
Single	20 (5.9)	19 (3.4)	0 (0.0)	
Separated/Widowed	79 (23.4)	9 (31.0)	2 (50.0)	
Education				
None	26 (7.7)	7 (24.1)	1 (25.0)	0.007 **
Some formal education	311 (92.3)	22 (75.9)	3 (75.0)	
Occupation				
Unemployed	99 (29.4)	10 (34.5)	3 (75.0)	0.125
Employed	238 (70.6)	19 (65.5)	1 (25.0)	
Family support				
No	78 (23.1)	5 (17.2)	3 (75.0)	0.037 *
Yes	259 (76.9)	24 (82.8)	1 (25.0)	
Diabetic complications				
Absent	60 (17.8)	7 (24.1)	0 (0.0)	0.446
Present	277 (82.2)	22 (75.9)	4 (100.0)	
Co-morbidity				
Absent	52 (15.4)	3 (10.3)	1 (25.0)	0.656
Present	285 (84.6)	26 (89.7)	3 (75.0)	
Duration of T2DM (in years)				
0–10	236 (70.0)	17 (58.6)	3 (75.0)	0.429
>10	101 (30.0)	12 (41.4)	1 (25.0)	
Type of diabetic management				
Lifestyle modification	7 (2.1)	0 (0.0)	0 (0.0)	0.504
Oral hypoglycemic drugs	321 (95.3)	27 (93.1)	4 (100.0)	
Insulin	5 (1.5)	2 (6.9)	0 (0.0)	
Insulin and oral hypoglycemic drugs	4 (1.2)	0 (0.0)	0 (0.0)	
HbA1c				
Good (<7)	82 (24.3)	7 (24.1)	1 (25.0)	0.764
Fair (7–8)	100 (29.7)	11 (37.9)	2 (50.0)	
Poor (>8)	155 (46.0)	11 (37.9)	1 (25.0)	

Fisher t-test; * *p* < 0.05, ** *p* < 0.01.

**Table 4:** Association between diabetes-related distress and potentially related factors (n = 370).

Characteristic	Total DRD	EB	PD	RD	ID
Age	0.891	0.682	0.704	0.583	0.199
Duration of T2DM (in years)	0.233	0.121	0.536	0.464	0.165
HbA1c	0.023 *	0.029 *	0.586	0.011 *	0.234
Co-morbidity	0.043 *	0.015 *	0.119	0.011 *	0.212

Pearson’s correlation coefficient; * *p* < 0.05 = statistically significant.

**Table 5:** Association between Hemoglobin A1c (HbA1c) and diabetes-related distress, using multiple linear regression analysis.

Characteristics	Regression Coefficient	SE	*p*-Value
HbA1c	0.645	0.300	0.032
Age	−0.015	0.051	0.769
Sex	−0.901	0.848	0.289
Education	−0.586	0.629	0.352
Duration of T2DM (year)	0.016	0.063	0.800
Co-morbidity	0.819	0.426	0.055
Diabetic complications	1.415	1.050	0.179
Family support	−1.356	0.912	0.138

Adjust for age, sex, education, duration of DM, co-morbidity, diabetic complications, family care.

## References

[1] Saeedi P., Petersohn I., Salpea P., Malanda B., Karuranga S., Unwin N., Colagiuri S., Guariguata L., Motala A.A., Ogurtsova K. (2019). Global and regional diabetes prevalence estimates for 2019 and projections for 2030 and 2045: Results from the International Diabetes Federation Diabetes Atlas. Diabetes Res. Clin. Pract..

[2] Chan J.C., Cho N.H., Tajima N., Shaw J. (2014). Diabetes in the Western pacific region—past, present and future. Diabetes Res. Clin. Pract..

[3] Aekplakorn W., Chariyalertsak S., Kessomboon P., Assanangkornchai S., Taneepanichskul S., Putwatana P. (2018). Prevalence of diabetes and relationship with socioeconomic status in the Thai population: National Health Examination Survey, 2004–2014. J. Diabetes Res..

[4] Reutrakul S., Deerochanawong C. (2016). Diabetes in Thailand: Status and policy. Curr. Diab. Rep..

[5] American Diabetes A. (2014). Diagnosis and classification of diabetes mellitus. Diabetes Care.

[6] Lotfy M., Adeghate J., Kalasz H., Singh J., Adeghate E. (2017). Chronic complications of diabetes mellitus: A mini review. Curr. Diabetes Rev..

[7] Association A.D. (2015). Standards of medical care in diabetes—2015: Summary of revisions. Diabetes Care.

[8] Luijks H., Schermer T., Bor H., van Weel C., Lagro-Janssen T., Biermans M., de Grauw W. (2012). Prevalence and incidence density rates of chronic comorbidity in type 2 diabetes patients: An exploratory cohort study. BMC Med..

[9] Sanal T.S., Nair N.S., Adhikar P. (2011). Factors associated with poor control of type 2 diabetes mel l i tus: A systematic review and Meta-analysis. J. Diabetol..

[10] Smith K.J., Page V., Gariepy G., Beland M., Badawi G., Schmitz N. (2012). Self-rated diabetes control in a Canadian population with type 2 diabetes: Associations with health behaviours and outcomes. Diabetes Res. Clin. Pract..

[11] Khattab M., Khader Y.S., Al-Khawaldeh A., Ajlouni K. (2010). Factors associated with poor glycemic control among patients with type 2 diabetes. J. Diabetes Complicat..

[12] Kellow N.J., Savige G.S., Khalil H. (2011). Predictors of poor glycaemic control during the initial five years post-diagnosis in rural adults with type 2 diabetes. Aust. J. Rural. Health.

[13] Juarez D.T., Sentell T., Tokumaru S., Goo R., Davis J.W., Mau M.M. (2012). Factors associated with poor glycemic control or wide glycemic variability among diabetes patients in Hawaii, 2006–2009. Prev. Chronic. Dis..

[14] Al-Hayek A.A., Robert A.A., Alzaid A.A., Nusair H.M., Zbaidi N.S., Al-Eithan M.H., Sam A.E. (2012). Association between diabetes self-care, medication adherence, anxiety, depression, and glycemic control in type 2 diabetes. Saudi Med. J..

[15] Kendzor D.E., Chen M., Reininger B.M., Businelle M.S., Stewart D.W., Fisher-Hoch S.P., Rentfro A.R., Wetter D.W., McCormick J.B. (2014). The association of depression and anxiety with glycemic control among Mexican Americans with diabetes living near the U.S.-Mexico border. BMC Public Health.

[16] Fisher L., Mullan J.T., Arean P., Glasgow R.E., Hessler D., Masharani U. (2010). Diabetes distress but not clinical depression or depressive symptoms is associated with glycemic control in both cross-sectional and longitudinal analyses. Diabetes Care.

[17] Tsujii S., Hayashino Y., Ishii H., Diabetes Distress and Care Registry at Tenri Study Group (2012). Diabetes distress, but not depressive symptoms, is associated with glycaemic control among Japanese patients with type 2 diabetes: Diabetes Distress and Care Registry at Tenri (DDCRT 1). Diabet Med..

[18] Fisher L., Glasgow R.E., Strycker L.A. (2010). The Relationship Between Diabetes Distress and Clinical Depression with Glycemic Control Among Patients with Type 2 Diabetes. Diabetes Care.

[19] Fisher L., Gonzalez J.S., Polonsky W.H. (2014). The confusing tale of depression and distress in patients with diabetes: A call for greater clarity and precision. Diabet Med..

[20] Thanakwang K., Thinganjana W., Konggumnerd R. (2014). Psychometric properties of the Thai version of the Diabetes Distress Scale in diabetic seniors. Clin. Interv. Aging.

[21] Polonsky W.H., Anderson B.J., Lohrer P.A., Welch G., Jacobson A.M., Aponte J.E., Schwartz C.E. (1995). Assessment of diabetes-related distress. Diabetes Care.

[22] Zhang J., Xu C.P., Wu H.X., Xue X.J., Xu Z.J., Li Y., Gao Q., Liu Q.Z. (2013). Comparative study of the influence of diabetes distress and depression on treatment adherence in Chinese patients with type 2 diabetes: A cross-sectional survey in the People’s Republic of China. Neuropsychiatr. Dis. Treat..

[23] Parsa S., Aghamohammadi M., Abazari M. (2019). Diabetes distress and its clinical determinants in patients with type II diabetes. Diabetes Metab. Syndr..

[24] Chew B.H., Vos R., Mohd-Sidik S., Rutten G.E. (2016). Diabetes-related distress, depression and distress-depression among adults with type 2 diabetes mellitus in Malaysia. PLoS ONE.

[25] Islam M.R., Karim M.R., Habib S.H., Yesmin K. (2013). Diabetes distress among type 2 diabetic patients. Int. J. Med. Biomed. Res..

[26] Fisher L., Skaff M.M., Mullan J.T., Arean P., Glasgow R., Masharani U.A. (2008). longitudinal study of affective and anxiety disorders, depressive affect and diabetes distress in adults with Type 2 diabetes. Diabet Med..

[27] Jia W., Gao X., Pang C., Hou X., Bao Y., Liu W., Wang W., Zuo Y., Gu H., Xiang K. (2009). Prevalence and risk factors of albuminuria and chronic kidney disease in Chinese population with type 2 diabetes and impaired glucose regulation: Shanghai diabetic complications study (SHDCS). Nephrol. Dial. Transpl..

[28] Ting R.Z., Nan H., Yu M.W., Kong A.P., Ma R.C., Wong R.Y., Loo K., So W.Y., Chow C.C., Ko G.T. (2011). Diabetes-related distress and physical and psychological health in Chinese type 2 diabetic patients. Diabetes Care.

[29] Fisher L., Mullan J.T., Skaff M.M., Glasgow R.E., Arean P., Hessler D. (2009). Predicting diabetes distress in patients with type 2 diabetes: A longitudinal study. Diabet. Med..

[30] Pandit A.U., Bailey S.C., Curtis L.M., Seligman H.K., Davis T.C., Parker R.M., Schillinger D., DeWalt D., Fleming D., Mohr D.C. (2014). Disease-related distress, self-care and clinical outcomes among low-income patients with diabetes. J. Epidemiol. Community Health.

[31] Aikens J.E. (2012). Prospective associations between emotional distress and poor outcomes in type 2 diabetes. Diabetes Care.

[32] Fisher L., Hessler D.M., Polonsky W.H., Mullan J. (2012). When Is Diabetes Distress Clinically Meaningful?: Establishing cut points for the Diabetes Distress Scale. Diabetes Care.

[33] Stankovic Z., Jasovic-Gasic M., Lecic-Tosevski D. (2013). Psychological problems in patients with type 2 diabetes―Clinical considerations. Vojn. Pregl..

[34] Conversano C. (2019). Psychological common factors in chronic diseases. Front. Psychol..

[35] Peyrot M., Rubin R.R. (2007). Behavioral and Psychosocial Interventions in Diabetes A conceptual review. Diabetes Care.

[36] Ngamjarus C., Chongsuvivatwong V., McNeil E. (2016). n4Studies: Sample size calculation for an epidemiological study on a smart device. SMJ.

[37] Polonsky W.H., Fisher L., Earles J., Dud R.J., Lees J., Mullan J., Jackson R.A. (2005). Assessing psychosocial distress in diabetes: Development of the diabetes distress scale. Diabetes Care.

[38] Zhou H., Zhu J., Liu L., Li F., Fish A.F., Chen T., Lou Q. (2017). Diabetes-related distress and its associated factors among patients with type 2 diabetes mellitus in China. Psychiatry Res..

[39] Kuniss N., Rechtacek T., Kloos C., Müller U.A., Roth J., Burghardt K., Kramer G. (2017). Diabetes-related burden and distress in people with diabetes mellitus at primary care level in Germany. Acta Diabetol..

[40] Kuniss N., Kramer G., Müller N., Kloos C., Lehmann T., Lorkowski S., Wolf G., Müller U.A. (2016). Diabetes-related burden and distress is low in people with diabetes at outpatient tertiary care level. Exp. Clin. Endocrinol. Diabetes.

[41] Aljuaid M.O., Almutairi A.M., Assiri M.A., Almalki D.M., Alswat K. (2018). Diabetes-related distress assessment among type 2 diabetes patients. J. Diabetes Res..

[42] Tol A., Baghbanian A., Sharifirad G., Shojaeizadeh D., Eslami A., Alhani F., Tehrani M.M. (2012). Assessment of diabetic distress and disease related factors in patients with type 2 diabetes in Isfahan: A way to tailor an effective intervention planning in Isfahan-Iran. J. Diabetes Metab. Disord..

[43] Stoop C.H., Nefs G., Pop V.J., Wijnands-van Gent C.J., Tack C.J., Geelhoed-Duijvestijn P.H., Diamant M., Snoek F.J., Pouwer F. (2014). Diabetes-specific emotional distress in people with Type 2 diabetes: A comparison between primary and secondary care. Diabet Med..

[44] Gahlan D., Rajput R., Gehlawat P., Gupta R. (2018). Prevalence and determinants of diabetes distress in patients of diabetes mellitus in a tertiary care centre. Diabetes Metab. Syndr..

[45] Karlsen B., Oftedal B., Bru E. (2012). The relationship between clinical indicators, coping styles, perceived support and diabetes-related distress among adults with type 2 diabetes. J. Adv. Nurs.

[46] Conti C., Carrozzino D., Patierno C., Vitacolonna E., Fulcheri M. (2016). The clinical link between type D personality and diabetes. Front. Psychiatry.

[47] Martino G., Bellone F., Langher V., Caputo A., Catalano A., Quattropani M.C., Morabito N. (2019). Alexithymia and Psychological Distress Affect Perceived Quality of Life in Patients with Type 2 Diabetes Mellitus. MJCP.

[48] Settineri S., Frisone F., Merlo E.M., Geraci D., Martino G. (2019). Compliance, adherence, concordance, empowerment, and self-management: Five words to manifest a relational maladjustment in diabetes. J. Multidiscip. Healthc..

[49] Nanayakkara N., Pease A., Ranasinha S., Wischer N., Andrikopoulos S., Speight J., de Courten B., Zoungas S. (2018). Depression and diabetes distress in adults with type 2 diabetes: Results from the Australian National Diabetes Audit (ANDA) 2016. Sci. Rep..

[50] Sankar P., Sasikumar P., Medayil R., Jacob R., Sasidharan S. (2018). High Prevalence of Distress among Patients with Type 2 Diabetes (T2DM)—A Hospital-Based Cross-Sectional Study from South India. Am. Diabetes Assoc..

[51] Ramkissoon C., Vehi J. Emotions and Diabetes. Proceedings of the International Conference on Bioinformatics and Biomedical Engineering.

